# Erectile Dysfunction in Men Burdened with the Familial Occurrence of Coronary Artery Disease

**DOI:** 10.3390/jcm10184046

**Published:** 2021-09-07

**Authors:** Dariusz Kałka, Jana Gebala, Małgorzata Biernikiewicz, Aneta Mrozek-Szetela, Krystyna Rożek-Piechura, Małgorzata Sobieszczańska, Ewa Szuster, Marzena Majchrowska, Anna Miętka, Agnieszka Rusiecka

**Affiliations:** 1 Cardiosexology Unit, Department of Physiology & Pathophysiology, Wrocław Medical University, 50-368 Wrocław, Poland; marzen89@gmail.com (M.M.); annbiel@wp.pl (A.M.); agnieszka.rusiecka@umed.wroc.pl (A.R.); 2 Centre for Men’s Health, 53-151 Wrocław, Poland; 3 Cardiosexology Students’ Scientific Club, Wrocław Medical University, 50-368 Wrocław, Poland; janagebala@aol.com (J.G.); ewa.szuster8@gmail.com (E.S.); 4 Studio Słowa, 50-357 Wrocław, Poland; malgorzata@biernikiewicz.pl; 5 Doctoral School at Wrocław of Environmental and Life Sciences, 50-375 Wrocław, Poland; vesta.vital@gmail.com; 6 Faculty of Physiotherapy, Wrocław University of Health and Sport Sciences, 51-612 Wrocław, Poland; krystyna.rozek-piechura@awf.wroc.pl; 7 Department of Geriatrics, Wrocław Medical University, 50-369 Wrocław, Poland; malsobie100@gmail.com

**Keywords:** cardiovascular disease, family health history, erectile dysfunction, physical activity, risk factors

## Abstract

Erectile dysfunction (ED) and coronary artery disease (CAD) share common risk factors, some of which have genetic backgrounds, while others may be stimulated by family lifestyle. We investigated the impact of the familial occurrence of CAD on the presence of ED and the presence of classic risk factors for ED in men with CAD. This cross-sectional observational study involved 751 men with CAD who were subjected to cardiac rehabilitation. Overall, 75.63% of the men had ED. CAD was diagnosed in 39.28% of the studied men’s relatives. ED was less frequent in the men with familial CAD than in those without (71.53% vs. 78.29%). Similar relations were observed for the presence of CAD in parents (70.43% vs. 78.34%) and the father (69.95% vs. 77.46%). The International Index of Erectile Function 5 score was significantly higher in patients with familial CAD (median (interquartile range); 17 (12–22) vs. 16 (10–21); *p* = 0.0118), in parents (18 (12–22) vs. 16 (10–20); *p* = 0.021), and in the father (18 (12–22) vs. 16 (10–21); *p* = 0.0499). Age and education minimized the effect of familial CAD. Familial CAD increased the incidence of hypertension, dyslipidemia, and smoking but not sedentary lifestyle. Despite the higher prevalence of selected risk factors for ED in men with familial CAD, a higher incidence of ED was not observed.

## 1. Introduction

A family history of coronary artery disease (CAD), a type of cardiovascular disease (CVD), has been thoroughly investigated and confirmed to be an independent risk factor for CAD in future generations [1]. Familial CAD increases the risk of developing CAD by 1.5–2.0, which draws attention to the role genetic factors play in the development of CAD in subsequent generations [2]. Evidence exists that the genes that regulate lipid metabolism, as well as those that control the activity of the renin–angiotensin–aldosterone (RAA) system, contribute to the development of CAD due to their impact on arterial blood pressure [2,3,4]. Apart from hereditary factors impacting the occurrence of CAD, family members also typically experience exposure to the same environmental factors, such as unhealthy behaviors like smoking and eating a diet with a high content of saturated fat.

A negative impact of genetic and environmental factors, which contribute to the pathogenesis of CAD, have a similar effect on the development of the whole spectrum of cardiovascular diseases, including vasculogenic erectile dysfunction (ED) [5,6]. The prevalence of ED increases with age. In the US, about 18 million men experience problems with sexual health [7], with CVD being the major cause of deaths [8]. Due to differences in physiology and anatomy, endothelial dysfunction and deterioration in penile blood flow appear much earlier than disorders in vessels in other parts of the arterial system. Thus, ED is considered to be an early risk factor for the development of CAD [9]. A history of coronary artery disease (CAD) in the family may influence the frequency of ED and erection quality due to its shared pathogenesis with ED. In the literature, however, there are no reports that would allow the evaluation of these associations and the stimulation of lifestyle changes that primarily aim to prevent ED in men burdened with familial CAD.

The aim of our study was to evaluate the impact of the familial occurrence of CAD on the presence of ED and the presence of classic risk factors for ED in men with CAD.

## 2. Materials and Methods

For this cross-sectional observational study, 751 patients with CAD, who were subjected to rehabilitative treatment, were recruited in 5 centers of cardiac rehabilitation. The characteristics of the study group are presented in Table 1.

All patients gave their written and voluntary consent for participation in the conducted study by completing a questionnaire. Respondents answered questions themselves, but they could explain any doubts with an interviewer. They were assured full confidentiality and were not influenced by any third party. The questionnaire included closed-ended questions. Collected data were coded, in turn providing all respondents with complete protection of their data.

The survey comprised questions on basic demographics; family history of CAD (in case of doubts—verified by medical records or by phone interview), including past myocardial infarction; percutaneous coronary interventions (PCI); and the implantation of coronary artery bypass grafts (CABG). Moreover, respondents answered questions on modifiable risk factors, the presence of which was confirmed by the analysis of medical records. The following factors were considered: smoking, hypertension, lipid disorders, diabetes, increased body weight, and health-promoting physical activity of low intensity (sedentary lifestyle). The intensity of physical activity was estimated using instructions modeled on the Framingham questionnaire, which allowed planned, intentional and regular forms of physical activity to be evaluated. Additionally, this category included commuting (either on foot or by bicycle), provided that it lasted longer than 15 min at a time. For the estimation of physical activity, the method of evaluation from the original Framingham questionnaire was used. The duration of activity periods was tracked with a precision of up to 15 min. Thousand kcal was set as the standard for the minimal intensity of leisure-time physical activity per week, as conducted to prevent primary diseases of the cardiovascular system [10,11].

The presence of ED was evaluated using an abridged International Index of Erectile Function 5 (IIEF-5) Questionnaire, which included 5 questions scored from 0 to 5 (the first question scored 1 to 5) for a total score of 5–25. ED was diagnosed if the overall score was ≤21, and the assessment was based on sexual intercourse during the last 6 months without stimulation with drugs that improve sexual performance [12]. Respondents reporting ED due to surgical treatment for anatomic abnormalities of the penis, prostatic hyperplasia or prostate cancer were excluded. Patients were also excluded who had a history of repair surgery of the abdominal aorta or iliac arteries, neurological treatment for any vascular event in the central nervous system, or orthopedic treatment for injuries to the spine or pelvis, as well as those undergoing current psychiatric supervision, antidepressive treatment, pulmonary therapy due to diseases that impair respiratory function, and hormone treatment with gonadotropin-releasing hormone (GnRH) agonists or androgens. The neurologic and orthopedic condition of patients did not affect motoric functioning and was not considered to be a contraindication for kinesitherapy.

The reliability of the IIEF-5 and Framingham questionnaires was verified by comparing the total score of the first test and the second control test (with at least a 7-day interval between the tests) in 85 randomly selected patients.

The study was conducted after obtaining approval from the Bioethics Committee at Wroclaw Medical University (KB-433/2010). The study is part of the PREVANDRO project. It serves as targeted cardiosexology education aimed at delivering knowledge on the risk factors for ED, the main symptoms of ED, the possible treatment options, and necessary lifestyle modifications to adhere to European guidelines on the prevention of cardiovascular diseases. The education program was conducted using a PowerPoint presentation, printed materials (leaflets, video, IIEF-5 questionnaire) and the website www.kardioseksuologia.pl (accessed date: March 2021). The program, conducted in the years 2011–2021, has reached 22,736 patients to date.

Data were statistically analyzed using the Statistica package v. 12 (StatSoft, Tulsa, OK, USA), and descriptive statistics were calculated. The Student’s t-test and the Mann-Whitney U test were used to compare numerical variables, and the Chi-square test (with the Yates correction for 2 × 2 tables) was used for dichotomous variables and trends for ordinal variables. Relationships were analyzed using Spearman’s rank correlation coefficient. Logistic regression models (generalized linear models) were used to determine the impact of family history on outcomes. Differences were considered to be statistically significant at a value of *p* < 0.05.

## 3. Results

In the study group, ED was present in 568 (75.63%) patients. It was associated with older age (61.59 ± 8.53 vs. 53.18 ± 9.15, *p* < 0.0001), lower education (*p* = 0.0451), diabetes (85.65% vs. 71.40%; *p* < 0.0001), a sedentary lifestyle (77.09% vs. 61.43%; *p* = 0.0057), a greater left ventricular diastolic dimension (54.43 ± 6.65 mm vs. 52.22 ± 6.24 mm; *p* = 0.0002), a greater left atrial dimension (42.15 ± 5.29 mm vs. 40.47 ± 4.36 mm; *p* = 0.0003), and a lower ejection fraction of the left ventricle (53.27 ± 9.83% vs. 56.21 ± 9.03%; *p* = 0.0008). The analysis of the used therapeutic options showed that ED occurred significantly more often in patients treated with CABG (83.76% vs. 70.38%, *p* < 0.0001), angiotensin II receptor blockers (93.10% vs. 74.17%; *p* = 0.0021), diuretics (81.95% vs. 72.16%; *p* = 0.0037), and alfa-blockers (93.94% vs. 74.79%; *p* = 0.0215). A lower occurrence of ED was associated with the use of angiotensin-converting-enzyme inhibitors (73.33% vs. 82.05%; *p* = 0.0192).

The IIEF-5 score was negatively correlated with age (R = −0.4346, *p* < 0.0001), the number of pack-years of smoking (R = −0.1995, *p* < 0.0001), the left ventricular diastolic dimension (R = −0.1654, *p* < 0.0001), and the left atrial dimension (R = −0.1547, *p* < 0.0001), while positively correlated with ejection fraction (R = 0.1277, *p* = 0.0010). A significantly lower score in the IIEF-5 questionnaire was associated with the presence of diabetes (median 15, IQR (interquartile range) (9–19) vs. median 17, IQR (12–22); *p* < 0.0001) and a sedentary lifestyle (median 16, IQR (11–21) vs. median 19, IQR (13–23); *p* = 0.0071). The analysis of the used treatments shows that a significantly higher score in the IIEF-5 questionnaire was associated with using angiotensin-converting-enzyme inhibitors (median 17; IQR (12–22) vs. median 15 (10–20); *p* = 0.0008), while a significantly lower score was associated with a history of CABG (median 15; IQR (9–19) vs. median 18 IQR (12–22), *p* < 0.0001), and also the use of angiotensin II receptor blockers (median 14, IQR (7–17) vs. median 17, IQR (11–22); *p* = 0.0004), calcium channel blockers (median 15; IQR (9–21) vs. median 17, IQR (11–21); *p* = 0.0297), diuretics (median 15, IQR (9–20) vs. median 17 (12–22); *p* = 0.0002), and alfa-blockers (median 13, IQR (10–16) vs. median 16 (11–22); *p* = 0.0085).

CAD was diagnosed in 39.28% of the relatives of the men from the study group. The number and percentage of first-degree relatives in a straight line and second-degree relatives in a collateral line who had CAD symptoms are depicted in Table 2. Familial CAD was associated with a lower percentage of ED when compared to men without a family history of CAD (71.53% vs. 78.29%). Similar relations were observed for the presence of CAD in parents (70.43% vs. 78.34%) and the father (69.95% vs. 77.46%). These relationships were no longer significant after adjusting for age and education.

The score in the IIEF questionnaire was significantly higher in patients with CAD in the family (median 17, IQR (12–22) vs. median 16, IQR (10–21)), in parents (median 18, IQR (12–22) vs. median 16, IQR (10–20)), and in the father (median 18, IQR (12–22) vs. median 16, IQR (10–21)). However, in these cases, the relationships were no longer significant after adjusting for age and education. *p*-values for the relationships are depicted in Table 3.

The presence of CAD in the family was associated (in the study group) with a more frequent occurrence of hypertension, lipid disorders, and a greater total number of risk factors for ED, as well as a significantly lower frequency of having a sedentary lifestyle. The presence of CAD in the parents was associated with a more frequent occurrence of hypertension and lipid disorders, as well as a significantly lower frequency of having a sedentary lifestyle. The presence of CAD in the father was associated with a more frequent occurrence of hypertension and a significantly lower frequency of having a sedentary lifestyle. The presence of CAD in the mother was associated with a more frequent occurrence of smoking, lipid disorders and a greater total number of risk factors for ED. The number and percentage of patients, along with *p*-values for associations of the occurrence of CAD in the family, parents, father, mother and risk factors for ED, are presented in Table 4.

Overall, the presence of CAD in the family increased the chance of hypertension (OR 1.745, 95%CI (1.213–2.511)) and lipid disorders (OR 1.553, 95%CI (1.144–2.107)), and decreased the chance of having a sedentary lifestyle (OR 1.959, 95%CI (1.194–3.214)). The presence of CAD in parents increased the chance for hypertension (OR 1.638, 95%CI (1.194–2.387)) and lipid disorders (OR 1.673, 95%CI (1.219–2.294)), and decreased the chance of having a sedentary lifestyle (OR 2.204, 95%CI (1.343–3.617)). The presence of CAD in the father increased the chance for hypertension (OR 1.854, 95%CI (1.195–2.876)), but also decreased the chance of having a sedentary lifestyle (OR 2.42, 95%CI (1.456–4.024)). The presence of CAD in the mother increased the chance for lipid disorders (OR 1.673, 95%CI (1.219–2.294)) and smoking (OR 1.952, 95%CI (1.081–3.523)) in the first place. The presence of CAD in the family was associated with a significantly greater expenditure on health-promoting physical activity (369.01 ± 443.12 vs. 302.84 ± 416.12; *p* = 0.0355). The same applies to the presence of CAD in parents (381.14 ± 447.80 vs. 298.24 ± 412.85; *p* = 0.0084).

## 4. Discussion

In our cohort of 751 male cardiac patients, ED was present in 75.63% of them. Such a high percentage of patients presenting with sexual dysfunction among patients with CAD is associated with a common aetiology of CAD and ED rooted in dysfunction of vascular endothelium. Due to the physiology of the erection process, in which the arterial supply depends to a large extent on the relaxation of the deep arteries of the corpora cavernosa, reaching 80%, the initial stage of damage to the vascular endothelium may lead to a deterioration of the filling of the corpora cavernosa and a lower hardness of the erection. Risk factors such as hypertension, dyslipidemia, smoking, diabetes, overweight and obesity, and a sedentary lifestyle have a significant and proven influence on the development of the dysfunction of the vascular endothelium. Some of these factors have proven genetic backgrounds, and some of them are associated with pathological behaviors, the source of which may already take place in the family home. Thus, the familial burden of atherosclerotic CAD may have a potential impact on the presence of risk factors and ED in future generations. In the studied group of patients, CAD was present in 39.28% of relatives.

The development of CAD may seem to be inevitable in the offspring of patients with CAD due to the genetic background of the disease, which in recent times has gained more and more attention. Moreover, cardiovascular diseases have a complex inheritance with an interplay among multiple genes and environmental factors. There are multiple disorders in which inherited DNA sequence variants increase the risk of disease development [3]. An additional impact of non-genetic factors and the association between genetic and environmental factors determine the final clinical picture and course of this disease.

Most cardiovascular risk traits show complex inheritance. Multiple genes, encoding enzymes and receptors are directly involved in the metabolism of the lipids and proteins that contribute to the development of CAD. For example, apolipoprotein B (apoB)—a component of low-density lipoprotein (LDL)—binds LDL with a specific receptor, which in turn enables LDL clearance from the circulation. A polymorphic variant of apoB (-516T alle) is associated with an elevated level of LDL cholesterol, increasing the risk of CAD [13]. Insertion-deletion polymorphism of the angiotensin-converting enzyme gene may significantly modify the onset and course of CAD through the RAA axis [14,15].

The etiology of arterial hypertension, which affects up to 20% of the overall population, is complex and multifactorial, and has an underlying genetic background. Some 100 variants of genes participate in the regulation of blood pressure, with each having only a small overall effect [16]. Up to 50% of the variability in blood pressure is rooted in hereditary factors, but the impact of environmental factors triggers the development of the phenotypic traits of the disease. Many mutations and gene polymorphisms have been described as predisposing genetic factors. They occur in genes that encode the angiotensin-converting enzyme, angiotensinogen polypeptide chain, mineralocorticoid receptor, and fibroblast growth factor type 1. However, in some phenotypes of hypertension, genetic mutations, which are often inherited in an autosomal dominant manner, play a major role, e.g., in pseudohypoaldosteronism type II (Gordon’s syndrome), apparent mineralocorticoid excess syndrome, and primary hyperaldosteronism type I and II [3,4,17].

In the analyzed group of patients, both dyslipidemia and arterial hypertension were associated with the presence of CAD in family history. The presence of CAD in the family, parents and father significantly increased the risk of hypertension by 1.745, 1.638, and 1.854 times, respectively. On the other hand, the presence of CAD in the family, parents and mother significantly increased the risk of dyslipidemia by 1.553, 1.673, and 1.855 times, respectively.

A family history of CAD is a recognized risk factor for the disease [1,2]. A study conducted on an American population of 1058 adults explored the association between family history of CAD and coronary risk factors. Patients were divided into 3 groups (a low, intermediate and high family history score), in which the impact of 60 potential risk factors was evaluated. Cholesterol, years of smoking, HDL concentration and triceps skinfold thickness remained significant risk factors after adjusting for age (*p* < 0.01). The authors concluded that there are other unknown risk factors that convey the effect of family history of CAD, and for this reason, family history should be considered as an independent risk factor for the disease [18]. Prospective nationwide surveys from Israel included 2690 patients with the first acute myocardial infarction. This study revealed that patients with a positive family history were younger (53 vs. 64 years), current smokers, predominantly men, and more often suffering from hyperlipidemia (yet less often suffering from diabetes and hypertension) in comparison to those with a negative family history. During hospitalization, patients with a positive family history developed heart failure less often, but received coronary angiography, coronary artery bypass grafting and percutaneous coronary more often. It was concluded that a better prognosis was associated with a family history of CAD, which was mostly due to the younger age of patients [19]. Prichard et al. noted that a family history of lifestyle-related chronic diseases could contribute to changes in lifestyle, which is due to the fact the provision of information on chronic disease risk encourages family members to change their lifestyle [20]. 

Among the risk factors associated with negative health behaviors in the studied group of patients, the presence of CAD in family history was associated with a higher incidence of smoking. The incidence of this destructive factor for the vascular endothelium was increasing in patients whose mothers had CAD. Its presence increased the incidence of addiction by 1952 times in the patients from the study group. The presence of CAD in family history also significantly influenced the presence of another cardioprotective risk factor, which is health-promoting physical activity. The presence of CAD in the family, parents and father significantly increased the chances of intensified health-promoting physical activity by 1.959, 2.204 and 2.42 times, respectively. Regardless of the fact that the presence of CAD in family history significantly increased the amount of weekly expenditure on health-promoting physical activity (369.01 ± 443.12 vs. 302.84 ± 416.12; *p* = 0.0355), the intensity of this exercise was too low, below the recommended level. Therefore, it cannot be considered a factor that had a beneficial effect on the health of patients from the study group.

Developmental patterns and family behaviors can contribute to the development of cardiovascular diseases and overlap inherited genetic risk factors. It has been revealed that family heritage contributes to the increase in the risk of CAD, but researchers are still investigating how parental CAD can impact the occurrence of cardiovascular diseases in their children. In addition to inherited factors, environmental factors can increase this risk. Jurj et al. examined 66,130 married couples and concluded that a shared marital environment causes similarities in the lifestyle of spouses and may contribute to comparable morbidity [21]. Active smokers expose their family members to passive smoking, in turn increasing the risk of heart disease among their family members. Furthermore, growing up in a smoking family increases the chances of becoming a smoker. A longitudinal, multigenerational study involving 1010 Americans showed that the risk of becoming a smoker is especially high among offspring living with a persistent heavy-smoking parent and a smoking older sibling. The status of family smoking was shown to be a predictor of the onset of daily smoking in children aged 14–15 years and of daily smoking in adolescents [22]. Family behaviors regarding health and diet are similarly transmitted from parents to children [23]. Moreover, the risk of developing obesity in later life can be modified in the antenatal period, which suggests the greater role of the mother in mediating this risk. Epidemiological studies showed that both overnutrition and malnutrition experienced by women during pregnancy result in increased rates of obesity, diabetes, and cardiovascular diseases in their offspring (metabolic intrauterine programming) [24].

The occurrence of sexual health problems is important in the case of cardiac patients. ED serves as a predictor of CVD, mainly due to the same shared risk factors and the common pathogenesis of both conditions [25]. The meta-analysis of prospective cohort studies has provided evidence that ED significantly increases the risk of CVD, CAD, stroke and all-cause mortality. The increase in the risk is independent of conventional risk factors for CVD [26]. The Multiethnic Study of Atherosclerosis revealed that ED is a significant predictor of severe cardiovascular events after adjustment for conventional risk factors for CVD, depression and the use of β-blockers [27]. To acknowledge the importance of ED for the assessment of cardiovascular risk, ED was included as an independent risk factor for CVD in the QRISK3 prediction algorithms in the UK [28].

Despite the significantly higher prevalence of risk factors in patients with familial CAD, both the incidence of ED and the level of the IIEF5 score did not differ significantly between the group with positive and negative family history of CAD after adjusting for key risk factors for ED such as age and education in the analysis. This may be because erectile function depends on many factors that were not taken into account in this study. Apart from the studied factors, the quality of sexual life depends on other biological factors, such as hormones or the condition of the nervous system, and psychological factors such as the emotional condition or the successful relationship. A positive aspect of this study is the information that the presence of CAD in family history does not have to be a factor that determines sexual health in subsequent generations of men.

## 5. Conclusions

Despite the higher prevalence of selected risk factors for ED in men with familial CAD, the more frequent incidence of ED, as well as significant differences in the IIEF5 scores in this group, were not observed.

## Figures and Tables

**Table 1:** Study group characteristics.

Variable	Total
No of patients, *n* (%)	751 (100)
Age, years	59.54 ± 9.40
Place of living, *n* (%)	
Rural area	9 (1.12)
Urban area	742 (98.88)
Education, *n* (%)	
Higher	145 (19.33)
Secondary	285 (38.00)
Vocational	270 (36.00)
Primary	50 (6.67)
Erectile dysfunction *, *n* (%)	
Severe (5–7 scores)	129 (22.71)
Moderate-to-severe (8–11 scores)	75 (13.20)
Moderate (12–16 scores)	182 (32.04)
Mild (17–21 scores)	182 (32.04)
No ED	183 (24.37)
Risk factors for erectile dysfunction, *n* (%)	
Arterial hypertension	575 (76.56)
Type II diabetes mellitus	223 (29.69)
Dyslipidemia	457 (60.85)
Tobacco smoking	599 (79.76)
Pack-years of smoking	35.22 ± 20.77
Active smoking	44 (5.86)
BMI, kg/m^2^	28.20 ± 4.32
Waist circumference	97.02 ± 10.26
Normal weight (BMI < 25 kg/m*2*)	149 (19.84)
Overweight (25 ≤ BMI < 30 kg/m^2^)	382 (50.87)
Obese (BMI ≥ 30 kg/m^2^)	220 (29.29)
Sedentary lifestyle (<1000 Kcal/week)	681 (90.68)
Mean intensity of leisure-time physical activity (kcal/week)	337.20 ± 431.34
Clinical data	
LVD, mm	53.87 ± 6.61
LA, mm	41.73 ± 5.12
EF, %	54.00 ± 9.72
RVD, mm	26.64 ± 5.06
Tolerance of effort, METs	7.0 (5.7—8.7)
Myocardial infarction, *n* (%)	564 (75.10)
PCI, *n* (%)	502 (66.84)
CABG, *n* (%)	315 (41.94)
PCI and CABG, *n* (%)	81 (10.79)
Conservative treatment, *n* (%)	15 (2.00)
Pharmacotherapy, *n* (%)	
Beta-blockers	708 (94.27)
Angiotensin-converting-enzyme inhibitors	555 (74.00)
Angiotensin II receptor blockers	58 (7.72)
Statins	711 (94.67)
Calcium channel blockers	133 (17.71)
Diuretics	266 (35.42)
Alfa-blockers	33 (4.39)

Results are displayed as *n* (%) or mean ± SD or median (interquartile range). * International Index of Erectile Function 5 ≤ 21 scores. CABG—coronary artery bypass graft; BMICbody mass index; ED—erectile dysfunction; EF—ejection fraction; LVD—left ventricular diameter; LA—left atrial diameter; MET—metabolic equivalent; PCI—percutaneous coronary intervention; RVD—right ventricular diameter.

**Table 2:** Number and percentage of first-degree relatives in a straight line and second-degree relatives in a collateral line who presented with coronary artery disease (CAD).

	Family	Parents	Father	Mother	Siblings
CAD	295 (39.28%)	257 (34.22%)	183 (24.37%)	113 (15.05%)	67 (9.72%)

CAD, coronary artery disease.

**Table 3:** *p*-values of associations between the occurrence of CAD and the presence of erectile dysfunction (ED) and IIEF-5 score crude and adjusted for age and education.

	Family	Parents	Father	Mother	Siblings
Erectile dysfunction	*p* = 0.0432 *p* = 0.3331 *	*p* = 0.0210 *p* = 0.2919 *	*p* = 0.0499 *p* = 0.3807 *	*p* = 0.6404	*p* = 0.5774
IIEF-5 questionnaire score	*p* = 0.0118 *p* = 0.0914 *	*p* = 0.0048 *p* = 0.0994 *	*p* = 0.0176 *p* = 0.2214 *	*p* = 0.2870	*p* = 0.5650

* *p* < 0.05 after adjusting for the patients’ age and education. CAD—coronary artery disease; ED—erectile dysfunction, IIEF-5—International Index of Erectile Function 5.

**Table 4:** Relationships between risk factors for erectile dysfunction and the occurrence of CAD in the family, parents, father, and mother.

Factor	Family	Parents	Father	Mother
Hypertension	82.37% vs. 72.81% 0.0033	82.10% vs. 73.68% 0.0126	84.15% vs. 74,12% 0.0072	0.299
BMI ≥ 25 kg/m^2^	0.8827	0.7763	0.9222	0.9249
Smoking	0.0868	0.1502	0.5912	87.61% vs. 78.37% 0.0334
Dyslipidaemia	67.12% vs. 56.80% 0.0059	68.09% vs. 57.09% 0.0043	0.0523	72.57% vs. 58.78% 0.0077
Leisure-time physical activity	87.12% vs. 92.98% 0.0101	85.99% vs. 93.12% 0.0022	84.15% vs. 92.78% 0.0008	0.2975
Diabetes	0.9874	0.5209	0.4750	0.0759
Total number of risk factors CAD	5 IQR (1–6) vs. 4 IQR (1–6) 0.0121	0.0607	0.2159	5 IQR (1–6) vs. 4 IQR (1–6) 0.0025

BMI—body mass index; CAD—coronary artery disease; IQR—interquartile range.

## Data Availability

Not applicable.
